# Efficacy of probiotics as adjuvant therapy in bronchial asthma: a systematic review and meta-analysis

**DOI:** 10.1186/s13223-024-00922-7

**Published:** 2024-11-19

**Authors:** Divya Balan, Tejaswini Baral, Mohan K. Manu, Aswini Kumar Mohapatra, Sonal Sekhar Miraj

**Affiliations:** 1 Department of Respiratory Medicine, Kasturba Medical College, Manipal, Manipal Academy of Higher Education, Manipal, Karnataka 576104 India; 2 Department of Pharmacy Practice, Manipal College of Pharmaceutical Sciences, Manipal Academy of Higher Education, Manipal, Karnataka 576104 India

**Keywords:** Asthma, Probiotics, Efficacy, Randomized controlled trials, Meta-analysis

## Abstract

**Background:**

Asthma is a chronic, heterogeneous disease characterized by airway inflammation. Asthma exacerbations significantly increase the disease burden, necessitating new therapeutic approaches. Emerging evidence suggests probiotics, through the gut-lung axis, may benefit asthma management by modulating immune responses and reducing inflammation.

**Methods:**

This systematic review and meta-analysis adhered to PRISMA guidelines and was registered with PROSPERO (CRD42023480098). A comprehensive search of PubMed, Scopus, Web of Science, and Embase was conducted up to March 2024. Inclusion criteria encompassed randomized controlled trials (RCTs) evaluating probiotic interventions in asthma patients. Statistical analysis was done using RevMan 5.3, with odds ratios (OR) and 95% confidence intervals (CI) calculated, and heterogeneity assessed using I^2^ statistics.

**Results:**

Twelve RCTs, comprising 1401 participants, met the inclusion criteria. The probiotic strains investigated included various Lactobacillus and Bifidobacterium species. Meta-analysis revealed significant improvements in asthma control test scores (OR 1.18, 95% CI: 1.18–3.64, p = 0.0001) following probiotic supplementation. Probiotics also improved fractional exhaled nitric oxide (FeNO) in one study, but pooled FeNO and eosinophil data were not statistically significant (p = 0.46 and p = 0.29, respectively). One study observed fewer asthma exacerbations in the probiotic group (24/212) compared to placebo (67/210), with no difference in exacerbation duration.

**Conclusion:**

Probiotic supplementation may be beneficial in improving asthma symptom control with no significant impact on lung function indices or eosinophil levels. Probiotics can be a potential adjunctive therapy in asthma management, particularly for asthma symptom control.

**Supplementary Information:**

The online version contains supplementary material available at 10.1186/s13223-024-00922-7.

## Introduction

Asthma is a heterogeneous disease characterized by chronic airway inflammation [1]. In 2019, there were an estimated 262 million prevalent cases of asthma globally, with an age-standardized prevalence of 3415.53 per 100,000 population [2]. The age-standardized mortality rate due to asthma globally in 2019 was 5.8 per 100,000 population [2]. Though symptoms of asthma get commonly resolved either spontaneously or through medication, certain patients may face exacerbations, which carry the potential for life-threatening outcomes [3]. These exacerbations markedly increase the disease's burden. One emerging area of interest is probiotics as a potential adjunctive therapy for asthma. Probiotics are "live microorganisms that, when administered in adequate amounts, confer a health benefit on the host" [4]. The gut-lung axis represents a bidirectional communication pathway between the gastrointestinal and respiratory systems [5]. Disruption of the gut microbiota composition, known as dysbiosis, has been implicated in the pathogenesis of various respiratory diseases, including asthma [5]. Understanding this axis offers new insights into disease pathogenesis and therapeutic interventions. Modulating the gut microbiome through probiotics and diet holds promise for various systemic conditions and improving overall health [6]. The mechanisms underlying the beneficial effects of probiotics in asthma are multifaceted. Probiotics can interact with the host immune system, promoting the production of anti-inflammatory cytokines and inhibiting the release of pro-inflammatory mediators [7].

Moreover, probiotics may strengthen the epithelial barrier in the gut and respiratory tract, reducing the translocation of allergens and pathogens and subsequently attenuating airway inflammation [7, 8]. A diverse array of probiotic strains, like *Lactobacillus* and *Bifidobacterium*, are under investigation for their potential role in asthma management [9, 10]. Research on lung microbiota suggests its transient nature in health, influenced by adjacent body sites and the environment. Gut-lung microbiota interactions impact respiratory health, with potential for novel therapies through improved understanding and functional guild identification via 'omics' approaches [5]. Recent randomized controlled trials show evidence that probiotics have a role in asthma control and reducing exacerbations [11, 12]. Hence, we conducted a systematic review and meta-analysis to study the beneficial impact of probiotics on asthma.

## Methodology

### Registration and protocol

This study followed the Preferred Reporting Items for Systematic Reviews and Meta-Analyses (PRISMA) guidelines for reporting the findings [13]*.* The study protocol was registered in PROSPERO: International Prospective Register of Systematic Reviews (registration number: CRD42023480098) before searching.

### Objectives

The research question for this systematic review is: 'What is the efficacy of probiotics in managing bronchial asthma?' The research question was deconstructed into the PICO framework, where 'population' refers to individuals diagnosed with bronchial asthma, irrespective of age, gender, or ethnicity. The 'intervention' entails the administration of probiotics, while the 'comparison' encompasses standard treatments or placebo. The primary 'outcome' is the efficacy and safety of probiotics in managing bronchial asthma. The systematic review aims to comprehensively evaluate the existing evidence regarding the potential benefits and risks associated with probiotic supplementation in individuals with bronchial asthma.

### Eligibility criteria

The studies were considered eligible to be included according to the following criteria.

Inclusion criteria: (1) Randomized controlled trials, (2) Individuals diagnosed with bronchial asthma, regardless of age, gender, ethnicity, or severity of the condition, (3) Studies involving the administration of probiotics as an intervention, regardless of the dosage forms (e.g., capsules, tablets, powders, fermented foods), (4) Studies with a comparison group, which may include a placebo, standard treatment for asthma, or no treatment (5) Studies reporting relevant outcome measures related to the efficacy and safety of probiotics in managing bronchial asthma, and (6) Studies published in any language with no restriction on publication date.

Exclusion criteria: (1) Studies involving probiotic interventions combined with other treatments or interventions unrelated to asthma management will be excluded, (2) Animal studies, (3) Non-retrievable articles or abstract-only papers.

### Information sources and search strategy

We have performed a systematic literature search to select eligible articles published in the electronic bibliographic databases from the establishment until March 2024. We systematically implemented an advanced electronic search in PubMed, Scopus, Web of Science, and Embase to search for eligible studies. The search strategy in the above database was performed using the keywords and medical subject headings (MeSH) terms ‘Probiotics’, ‘Bronchial asthma’, and ‘Probiotic agent’ using ‘AND’ and ‘OR.’ We limited the search to English publications.

### Study selection process and data extraction

The studies were screened by title and abstracts followed by full-text articles based on predefined criteria. Two independent reviewers (D.B. and T.B.) performed the study selection, and disagreements were resolved by mutual consultation with a third reviewer (M.K.M). A well-defined data extraction sheet was employed. Data from the final selected studies included authors’ names, year of publication, study design, sample size, study groups, and clinical outcomes. One reviewer (T.B.) extracted the data in a standardized extraction sheet, and the other reviewer (D.B.) checked for accuracy. Discussions or consultations with a third reviewer (M.K.M) resolved disagreements.

### Risk of bias assessment

The Cochrane risk of bias (CROB) assessment tool was used to assess the methodological quality of the included studies***.*** Two independent reviewers (D.B. and T.B.) performed the quality assessment, and any disagreements were settled through consensus or discussion with a third reviewer (M.K.M.).

### Data synthesis

All the extracted information about the study was synthesized qualitatively and presented narratively. The data extracted from the included studies are represented in tabular form. The data synthesized in this review summarized the efficacy of current evidence for probiotic intervention supplementation in asthma.

### Statistical analysis

We conducted a meta-analysis using Review Manager Software (RevMan, version 5.3 for Windows; The Cochrane Collaboration, Oxford, UK). Odds ratios (OR) with 95% confidence intervals (CI) were computed. To evaluate statistical heterogeneity, we have used the I^2^ statistics. Studies were subjected to the fixed-effects model if no significant heterogeneity was observed (I^2^ ≤ 50% or P ≥ 0.10).

## Results

### Study selection

**Fig. 1:**
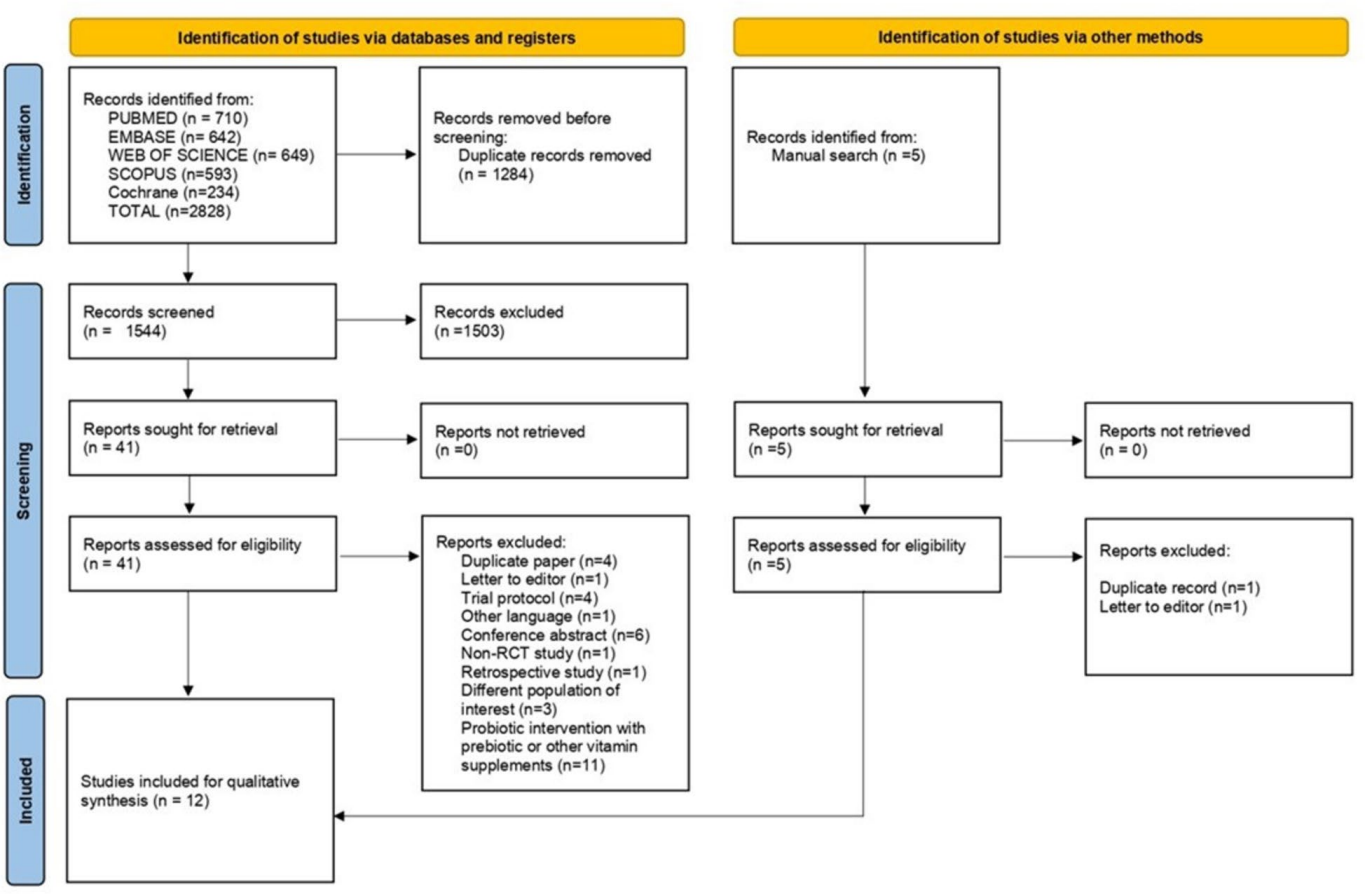
PRISMA flow diagram of the screening and selection process

A total of 2828 records have been identified after a systematic literature search from the abovementioned databases. Prior to the title and abstract screening, 1284 duplicate records were removed. Subsequently, 41 full-text articles were retrieved. Five articles were retrieved from a manual search as well. Full-text screening was performed on 46 articles based on our review eligibility criteria. Finally, 12 studies were incorporated into our review [11, 12, 14–23] (see Fig. 1).

### Study characteristics

All 12 studies included were placebo-controlled randomized trials. The types of probiotic intervention used have been mentioned in Table 1.

**Table 1:** Characteristics of included studies

Study	Type of probiotic intervention	Number of participants in the intervention group	Number of participants in the placebo group	Duration of intervention
Li et al. 2022*[14]	*Lactobacillus reuteri CCFM1040*	4	4	8 weeks
Drago et al. 2022[11]	*Ligilactobacillus salivarius LS01 (DSM 22775), Bifidobacterium breve B632 (DSM 24706)*	212	210	16 weeks
Liu et al. 2021[12]	*Bifidobacterium lactis Probio-M8*	30	26	3 months
Satia 2021[15]	*Limosilactobacillus reuteri DSM-17938*	114		4 weeks
Huang et al. 2018**[16]	*Lactobacillus paracasei, Lactobacillus fermentum &* both of their combination	114	38	3 months
Giudice et al. 2017[17]	*Bifidobacterium longum BB536, Bifidobacterium infantis M-63, Bifidobacterium breve M-16 V*	20	20	2 months
Liu et al. 2016[18]	*Clostridium butyricum*	15	14	6 months
Smith et al. 2016[19]	*Lactobacillus acidophilus CUL60 (NCIMB 30157)* and *CUL21 (NCIMB 30156), Bifidobacterium bifidum CUL20 (NCIMB 30153)* and* B. animalis (var lactis) CUL34 (NCIMB 30172)*	652	632	6 months
Chen et al. 2010[20]	*Lactobacillus gasseri PM-A0005*	55	56	8 weeks
Gutkowski et al. 2010[21]	*Lactobacillus acidophilus, Bifidobacterium bifidrum and Lactobacillus delbrueckii subsp. Bulgaricus*	22	24	12 weeks
Rose et al. 2010 [22]	*Lactobacillus rhamnosus*	65	46	12 weeks
Giovannini et al. 2007[23]	*Lactobacillus bulgaricus, Streptococcus thermophilus, and Lactobacillus casei DN-114 001*	98	77	6 months

*Values are taken for only asthma patients

**Huang 2018 has three intervention arms of two types of probiotics and their combination

### Asthma exacerbation

Drago et al. 2022, reported a reported a significantly reduced proportion of patients in the probiotic group who experienced asthma exacerbations, with 24 out of 212 patients in the probiotic group and 67 out of 210 in the placebo group (11). However, the mean asthma exacerbation duration was similar in both groups, which were 3.3 ± 2.45 days and 3.3 ± 2.57 days in the probiotic and placebo groups, respectively. They have used a multi-strain probiotics formulation of *Ligilactobacillus salivarius LS01* and *Bifidobacterium breve B632*.

### Asthma control test

Of all the included studies, Liu et al. 2021 and Li et al. 2022 reported asthma control test scores after the probiotic intervention [12, 14]. Both studies showed improved asthma control test scores after the probiotic intervention. *Li *et al. *2022* used *Lactobacillus reuteri CCFM1040* as an intervention, whereas *Liu *et al. *2021* used *Bifidobacterium lactis Probio-M8* as their study intervention [12, 14].

**Fig. 2:**
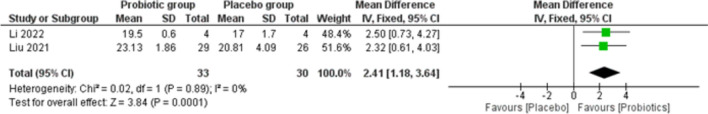
Forest plot for asthma control test

The fixed effect model was chosen due to the absence of statistical heterogeneity among studies (I^2^ ≤ 50% or P ≥ 0.10). The pooled analysis of two studies with 63 asthma patients showed a significant p-value of 0.0001 and an OR of 1.18 with a 95% CI: 1.18–3.64. (Fig. 2).

### Airway inflammation biomarker

**Fig. 3:**
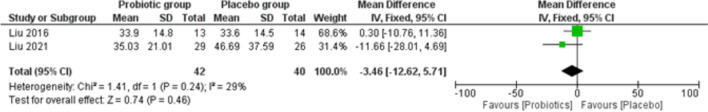
Forest plot for FeNO

Out of all the studies included, we could extract the data on fractional exhaled nitric oxide (FeNO) from Liu et al. 2016 and Liu et al. 2021 [18, 24]*.* While Liu et al. 2016 did not conclude any significant impact of the probiotic intervention on FeNO, Liu et al. 2021 concluded that the probiotic-treated group improved FeNO compared to the placebo group [18, 24]. However, the pooled analysis revealed statistically non-significant (p-value of 0.46) (Fig. 3).

**Fig. 4:**
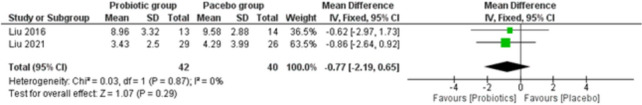
Forest plot for eosinophils

Liu et al. 2016 found lower eosinophils at six months of *Clostridium butyricum* intervention than the placebo group [18]. Likewise, Liu et al. 2021 observed with *Bifidobacterium lactis Probio-M8* intervention [24]. However, from pooled analysis, we could not find a statistically significant (p-value of 0.29) impact of probiotics intervention on the eosinophilia condition (Fig. 4).

### Lung function indices

Out of all the studies included, we could extract the data on forced expiratory volume in 1 s (FEV1) from Liu et al. 2016, Liu et al. 2021, Chen et al. 2010, and Gutkowski et al. 2010, none of the studies concluded any significant impact of probiotic intervention on FEV1. Since the data units were not uniform across the studies a meta-analysis could not be performed. [12, 18, 20, 21]

We also extracted data on peak expiratory flow rate (PEFR) from Liu et al. 2016, Liu et al. 2021 and Chen et al. 2010. All three studies found no significant impact of probiotic intervention on PEFR. [12, 18, 20]

### Adverse effects

Out of all the studies included, we could extract data on adverse effects from Li et al. 2022, Drago et al. 2022, Satia et al. 2021, Huang et al. 2018, Smith et al. 2016, Chen et al. 2010. Only Satia et al. 2021 reported that one patient developed a mild upper respiratory tract infection during the treatment period and one subject developed mild transient nausea with no serious adverse events. However, no adverse effects were reported with probiotics intervention in other studies [11, 14–16, 19, 20]

### Risk of bias assessment

We have graded the risk of bias assessment as low, high, and unclear by using the CROB tool (Table 2).

**Table 2:** Risk of bias assessment of all studies included

Study (Ref)	Sequence generation	Allocation concealment	Blinding of participants and personnel	Blinding of outcome assessment	Incomplete outcome data	Selective reporting	Other bias
Li et al. 2022 [14]	Low	Low	Unclear	Unclear	Low	Low	Unclear
Drago et al. 2022 [11]	Low	Low	Low	Low	Low	Low	Low
Liu et al. 2021 [12]	Unclear	Unclear	Low	Low	Low	Low	Low
Satia et al. 2021 [15]	Low	Low	Low	Low	Low	Unclear	Low
Huang et al. 2018 [16]	Low	Low	Low	Low	Low	Low	Low
Giudice et al. 2017 [17]	Low	Low	Low	Low	Low	Unclear	Low
Liu et al. 2016 [18]	Unclear	Low	Unclear	Unclear	Low	Low	Unclear
Smith et al. 2016 [19]	Low	Low	Low	Low	Low	Unclear	Low
Chen 2010 [20]	Low	Low	Low	Low	Low	Low	Low
Gutkowski et al. 2010 [21]	Unclear	Unclear	Low	Low	Low	Low	Unclear
Rose et al. 2010 [22]	Unclear	Low	Low	Low	Low	Unclear	Low
Giovannini et al. 2007 [23]	Low	Low	Low	Low	Low	Unclear	Low

## Discussion

Our systematic review included 12 studies of 1401 asthmatic patients who received probiotics as adjuvant therapy. We observed various probiotic strains used in asthma treatment, among which the most common probiotic genus was *Lactobacillus* and *Bifidobacterium*.

Several strains of *Lactobacillus* have been studied for their potential benefits in asthma. *Lactobacillus rhamnosus*, *Lactobacillus reuteri*, and *Lactobacillus gasseri* are among the most investigated strains [9, 25, 26]. Studies have shown that *Lactobacillus* can modulate immune responses and reduce airway inflammation in the context of allergic asthma [9, 25, 26]. *Lactobacillus* species and their components (peptidoglycan etc.) enhance respiratory health via complex mechanisms. The gut-lung axis is critical, where oral probiotics activate immune cells and cytokines in the gut, migrating to the lungs to boost immunity [27]. *Bifidobacteria* are the predominant inhabitants of the human gut microbiota, playing a crucial role in gut homeostasis and immune function [11]. *Bifidobacterium breve, Bifidobacterium lactis, and Bifidobacterium longum* are among the most studied strains of asthma [11, 28]. *Bifidobacteria* have been shown to enhance mucosal barrier function, stimulate the production of anti-inflammatory cytokines, and regulate T-cell responses, thereby exerting immunomodulatory effects that may benefit asthma patients [10].

From meta-analysis, we found that probiotic supplementation is associated with improvements in asthma control. Our pooled analysis supports the efficacy of probiotics in improving asthma control test scores. Despite the use of different probiotic strains (*Lactobacillus reuteri CCFM1040* and *Bifidobacterium lactis Probio-M8*), the observed improvements suggest probiotics can modulate the host immune system and maintain the microbial balance in the gut [7]. It modifies the host's Th1/Th2 balance by producing cytokines that enhance the Th1 pathway and suppress the Th2 pathway, which is associated with asthma and other allergic conditions [29].

Enhanced asthma control is associated with reduced symptom burden, decreased risk of exacerbations, and improved quality of life for individuals with asthma [1]. Probiotics may be a valuable adjunctive therapy for optimizing asthma management and improving patient outcomes, particularly for individuals with suboptimal disease control. The absence of statistical heterogeneity among the studies suggests a consistent effect of probiotics on asthma control, despite differences in probiotic strains used.

The meta-analysis revealed no significant impact of probiotics on T2-inflammation biomarkers and blood eosinophil level reduction. Factors such as participant characteristics, probiotic strain type, and intervention durations might have influenced the overall findings of the pooled analysis.

We observed no adverse drug reactions associated with probiotics as adjuvant therapy. The diversity of probiotic strains used across studies reflects the wide range of commercial microbial species being explored for their potential benefits in asthma management [11, 14–24]. It demonstrates the complexity of the gut-lung axis and the potential for various microbial species to influence immune responses and airway inflammation in individuals with asthma.

We acknowledge that there are not many studies on any specific probiotic strain for treating asthma from where we could have narrowed it down to a particular strain for the meta-analysis. Therefore, we warrant that more controlled trials are needed to investigate specific probiotic strains’ potential role in reducing asthma exacerbations and improving asthma control. The robust evidence in this area could find the place of probiotics adjuvant therapy in asthma management guidelines.

## Conclusion

This systematic review highlights the potential of probiotics in managing asthma by reducing exacerbations and improving asthma control. Evidence suggested significant improvements in asthma control test scores and a potential reduction in exacerbation rates, with no significant impact on lung function. Although there were trends toward reduced eosinophil levels, the pooled analysis lacked statistical significance, and no adverse events were reported. Therefore, comparative studies on strain-specific efficacy are required.

## Supplementary Information

Supplementary Material 1.

## Data Availability

No datasets were generated or analysed during the current study.

## Abbreviations

PRISMA: Preferred Reporting Items for Systematic Reviews and Meta-Analyses
PROSPERO: International Prospective Register of Systematic Reviews
MeSH: Medical subject headings
CROB: Cochrane risk of bias
OR: Odds ratios
CI: Confidence interval
FEV1: Forced expiratory volume in 1 s
PEFR: Peak expiratory flow rate

## References

[1] Global Initiative for Asthma - GINA. 2023 GINA Main Report, 2023. https://ginasthma.org/2023-gina-main-report/; Accessed 15 Apr 2024.

[2] Wang Z, Li Y, Gao Y, Fu Y, Lin J, Lei X, et al. Global, regional, and national burden of asthma and its attributable risk factors from 1990 to 2019: a systematic analysis for the Global Burden of Disease Study 2019. Respir Res. 2023;24:169. 37353829 10.1186/s12931-023-02475-6 PMC10288698

[3] Zeitouni MO, Al-Moamary MS, Coussa ML, Riachy M, Mahboub B, AlHuraish F, et al. Challenges and recommendations for the management of asthma in the Middle East and Africa. Ann Thorac Med. 2022;17:71–80. 35651897 10.4103/atm.atm_469_21 PMC9150662

[4] Hill C, Guarner F, Reid G, Gibson GR, Merenstein DJ, Pot B, et al. Expert consensus document. The International Scientific Association for Probiotics and Prebiotics consensus statement on the scope and appropriate use of the term probiotic. Nat Rev Gastroenterol Hepatol. 2014;11:506–14. 24912386 10.1038/nrgastro.2014.66

[5] Budden KF, Gellatly SL, Wood DLA, Cooper MA, Morrison M, Hugenholtz P, et al. Emerging pathogenic links between microbiota and the gut–lung axis. Nat Rev Microbiol. 2017;15:55–63. 27694885 10.1038/nrmicro.2016.142

[6] Quaranta G, Guarnaccia A, Fancello G, Agrillo C, Iannarelli F, Sanguinetti M, et al. Fecal microbiota transplantation and other gut microbiota manipulation strategies. Microorganisms. 2022;10:2424. 36557677 10.3390/microorganisms10122424 PMC9781458

[7] Sadrifar S, Abbasi-Dokht T, Forouzandeh S, Malek F, Yousefi B, Salek Farrokhi A, et al. Immunomodulatory effects of probiotic supplementation in patients with asthma: a randomized, double-blind, placebo-controlled trial. Allergy Asthma Clin Immunol Off J Can Soc Allergy Clin Immunol. 2023;19:1. 10.1186/s13223-022-00753-4 PMC9806812 36593510

[8] Maldonado Galdeano C, Cazorla SI, Lemme Dumit JM, Vélez E, Perdigón G. Beneficial effects of probiotic consumption on the immune system. Ann Nutr Metab. 2019;74:115–24. 30673668 10.1159/000496426

[9] Spacova I, Van Beeck W, Seys S, Devos F, Vanoirbeek J, Vanderleyden J, et al. Lactobacillus rhamnosus probiotic prevents airway function deterioration and promotes gut microbiome resilience in a murine asthma model. Gut Microbes. 2020;11:1729–44. 32522072 10.1080/19490976.2020.1766345 PMC7524350

[10] Sangkanjanavanich S, Pradubpongsa P, Mitthamsiri W, Sangasapaviliya A, Boonpiyathad T. Bifidobacterium infantis 35624 efficacy in patients with uncontrolled asthma: a randomized placebo-controlled trial. Ann Allergy Asthma Immunol. 2022;129:790–2. 36089255 10.1016/j.anai.2022.08.1000

[11] Drago L, Cioffi L, Giuliano M, Pane M, Amoruso A, Schiavetti I, et al. The probiotics in pediatric asthma management (PROPAM) study in the primary care setting: a randomized, controlled, double-blind trial with *Ligilactobacillus salivarius* LS01 (DSM 22775) and Bifidobacterium breve B632 (DSM 24706). J Immunol Res. 2022;2022:3837418. 35083341 10.1155/2022/3837418 PMC8786459

[12] Liu A, Ma T, Xu N, Jin H, Zhao F, Kwok LY, et al. Adjunctive probiotics alleviates asthmatic symptoms via modulating the gut microbiome and serum metabolome. Microbiol Spectr. 2021;9:e00859-e921. 34612663 10.1128/Spectrum.00859-21 PMC8510161

[13] PRISMA statement, 2020. https://www.prisma-statement.org/; Accessed 29 Apr 2024.

[14] Li L, Fang Z, Lee Y, Zhao J, Zhang H, Peng H, et al. Efficacy and safety of lactobacillus reuteri ccfm1040 in allergic rhinitis and asthma: a randomized. Placebo-Controlled Trial Front Nutr. 2022;9:862934. 35464005 10.3389/fnut.2022.862934 PMC9022948

[15] Satia I, Cusack R, Stevens C, Schlatman A, Wattie J, Mian F, et al. Limosilactobacillus reuteri DSM-17938 for preventing cough in adults with mild allergic asthma: a double-blind randomized placebo-controlled cross-over study. Clin Exp Allergy. 2021;51:1133–43. 34192396 10.1111/cea.13976

[16] Huang CF, Chie WC, Wang IJ. Efficacy of *Lactobacillus* administration in school-age children with asthma: a randomized. Placebo-Controlled Trial Nutrients. 2018;10:1678. 30400588 10.3390/nu10111678 PMC6265750

[17] Miraglia Del Giudice M, Indolfi C, Capasso M, Maiello N, Decimo F, Ciprandi G. Bifidobacterium mixture (B longum BB536, B infantis M-63, B breve M-16V) treatment in children with seasonal allergic rhinitis and intermittent asthma. Ital J Pediatr. 2017;43:25. 28270216 10.1186/s13052-017-0340-5 PMC5341466

[18] Liu J, Chen F, Qiu SQ, Yang LT, Zhang HP, Liu JQ, et al. Probiotics enhance the effect of allergy immunotherapy on regulating antigen specific B cell activity in asthma patients. Am J Transl Res. 2016;8:5256–70. 28078000 PMC5209480

[19] Smith TDH, Watt H, Gunn L, Car J, Boyle RJ. Recommending oral probiotics to reduce winter antibiotic prescriptions in people with asthma: a pragmatic randomized controlled trial. Ann Fam Med. 2016;14:422–30. 27621158 10.1370/afm.1970 PMC5394362

[20] Chen YS, Lin YL, Jan RL, Chen HH, Wang JY. Randomized placebo-controlled trial of lactobacillus on asthmatic children with allergic rhinitis. Pediatr Pulmonol. 2010;45:1111–20. 20658483 10.1002/ppul.21296

[21] Gutkowski P, Madaliński K, Grek M, Dmeńska H, Syczewska M, Michałkiewicz J. Effect of orally administered probiotic strains *Lactobacillus* and *Bifidobacterium* in children with atopic asthma. Cent Eur J Immunol. 2011;35:233–8.

[22] Rose MA, Stieglitz F, Köksal A, Schubert R, Schulze J, Zielen S. Efficacy of probiotic *Lactobacillus GG* on allergic sensitization and asthma in infants at risk. Clin Exp Allergy. 2010;40:1398–405. 20604800 10.1111/j.1365-2222.2010.03560.x

[23] Giovannini M, Agostoni C, Riva E, Salvini F, Ruscitto A, Zuccotti GV, et al. A randomized prospective double blind controlled trial on effects of long-term consumption of fermented milk containing lactobacillus casei in pre-school children with allergic asthma and/or rhinitis. Pediatr Res. 2007;62:215–20. 17597643 10.1203/PDR.0b013e3180a76d94

[24] Liu A, Ma T, Xu N, Jin H, Zhao F, Kwok LY, et al. Adjunctive probiotics alleviates asthmatic symptoms via modulating the gut microbiome and serum metabolome. Microbiol Spectr. 2021;9: e0085921. 34612663 10.1128/Spectrum.00859-21 PMC8510161

[25] Forsythe P, Inman MD, Bienenstock J. Oral treatment with live lactobacillus reuteri inhibits the allergic airway response in mice. Am J Respir Crit Care Med. 2007;175:561–9. 17204726 10.1164/rccm.200606-821OC

[26] Chen PC, Hsieh MH, Kuo WS, Wu LS, Kao HF, Liu LF, et al. Moonlighting glyceraldehyde-3-phosphate dehydrogenase (GAPDH) protein of Lactobacillus gasseri attenuates allergic asthma via immunometabolic change in macrophages. J Biomed Sci. 2022;29:75. 36175886 10.1186/s12929-022-00861-8 PMC9520948

[27] Du T, Lei A, Zhang N, Zhu C. The beneficial role of probiotic lactobacillus in respiratory diseases. Front Immunol. 2022;13: 908010. 35711436 10.3389/fimmu.2022.908010 PMC9194447

[28] Ciprandi G, Tosca MA, Drago L. Probiotics in asthma management: fiction or truth? Expert Rev Clin Immunol. 2023;19:457–60. 37094604 10.1080/1744666X.2023.2189103

[29] Zuccotti G, Meneghin F, Aceti A, Barone G, Callegari ML, Di Mauro A, et al. Italian Society of Neonatology. Probiotics for prevention of atopic diseases in infants: systematic review and meta-analysis. Allergy. 2015;70:1356–71. 26198702 10.1111/all.12700

